# The *Rhizobium tropici* CIAT 899 NodD2 protein promotes symbiosis and extends rhizobial nodulation range by constitutive nodulation factor synthesis

**DOI:** 10.1093/jxb/erac325

**Published:** 2022-07-28

**Authors:** Paula Ayala-García, Irene Jiménez-Guerrero, Catherine N Jacott, Francisco Javier López-Baena, Francisco Javier Ollero, Pablo del Cerro, Francisco Pérez-Montaño

**Affiliations:** Departamento de Microbiologia, Facultad de Biologia, Universidad de Sevilla, Seville, Spain; Departamento de Microbiologia, Facultad de Biologia, Universidad de Sevilla, Seville, Spain; Department of Crop Genetics, John Innes Centre, Norwich Research Park, Norwich, UK; Departamento de Microbiologia, Facultad de Biologia, Universidad de Sevilla, Seville, Spain; Departamento de Microbiologia, Facultad de Biologia, Universidad de Sevilla, Seville, Spain; Departamento de Microbiologia, Facultad de Biologia, Universidad de Sevilla, Seville, Spain; Departamento de Microbiologia, Facultad de Biologia, Universidad de Sevilla, Seville, Spain; University of Tasmania, Australia

**Keywords:** Common bean, infection thread, nodulation, nodulation factor, *Lotus*, rhizobium–legume symbiosis, *Rhizobium tropici*

## Abstract

In the symbiotic associations between rhizobia and legumes, the NodD regulators orchestrate the transcription of the specific nodulation genes. This set of genes is involved in the synthesis of nodulation factors, which are responsible for initiating the nodulation process. *Rhizobium tropici* CIAT 899 is the most successful symbiont of *Phaseolus vulgaris* and can nodulate a variety of legumes. Among the five NodD regulators present in this rhizobium, only NodD1 and NodD2 seem to have a role in the symbiotic process. However, the individual role of each NodD in the absence of the other proteins has remained elusive. In this work, we show that the CIAT 899 NodD2 does not require activation by inducers to promote the synthesis of nodulation factors. A CIAT 899 strain overexpressing *nodD2*, but lacking all additional *nodD* genes, can nodulate three different legumes as efficiently as the wild type. Interestingly, CIAT 899 NodD2-mediated gain of nodulation can be extended to another rhizobial species, since its overproduction in *Sinorhizobium fredii* HH103 not only increases the number of nitrogen-fixing nodules in two host legumes but also results in nodule development in incompatible legumes. These findings potentially open exciting opportunities to develop rhizobial inoculants and increase legume crop production.

## Teaser

*nodD2*overexpression in a quintuple *nodD*mutant of *Rhizobium tropici*CIAT 899 constitutively promotes Nod factor production, fully restoring nodulation ability with three different legumes, and also extends nodulation range in another rhizobial strain.

## Introduction

Nitrogen is an important macronutrient essential for all aspects of plant growth and crop productivity (White and Brown, 2010). Paradoxically, nitrogen has poor availability in soils leading to extensive fertilizer synthesis and application onto agricultural soils: both are highly energy-demanding processes that contribute to climate change and soil pollution (Tilman *et al.*, 2002; Woods *et al.*, 2010; Iyngaran *et al.*, 2011). Legume plants can establish a symbiotic interaction with certain soil proteobacteria—known as rhizobia—that convert atmospheric nitrogen to ammonia inside specialized root organs called nodules, thereby increasing nitrogen availability to the host plant and improving plant performance (Oldroyd, 2013). Thus, the application of nitrogen-fixing bacteria to legume crops presents an opportunity for sustainable agriculture.

Establishing the legume–rhizobium symbiotic interaction requires a complex and evolved molecular exchange of signals between both organisms. Compatible flavonoids exuded from legume roots into the rhizosphere are recognized by rhizobial NodD transcriptional regulators (Cooper, 2007; Hassan and Mathesius, 2012). The flavonoid-activated NodD protein then binds to conserved promoter regions upstream of nodulation (*nod*) genes, activating their expression (Schlaman *et al.*, 1998; Peck *et al.*, 2006; Long, 2016). The Nod proteins encoded by these genes are involved in the synthesis and export of lipochitooligosaccharides, also known as Nod factors (NF). In exchange, NFs are perceived by specific plant LysM-receptors that trigger a downstream signalling cascade leading to rhizobium-specific intracellular colonization of the root hair via infection thread formation and eventual development of nodules on roots (Dénarié *et al.*, 1996; Radutoiu *et al.*, 2003). An ancient and less sophisticated mechanism of rhizobial intercellular root infection via cracks at emergent lateral roots or between epidermal cells is also widespread in the legume family (Sprent *et al.*, 2007; Montiel *et al.*, 2021).

Recent studies suggest that NodD proteins may have additional roles beyond post-initial recognition and that flavonoid-independent inducers may activate NodD. For example, *Mesorhizobium loti* R7A NodD2 is active in the nodules, unlike NodD1 which is active during the root-hair colonization at the infection thread (Kelly *et al.*, 2018). Interestingly, *Rhizobium leguminosarum* sv. *trifolii* strains that harbour a second NodD copy exhibit enhanced nodule colonization and competitiveness (Ferguson *et al.*, 2020). In both *M. loti* and *R. leguminosarum* sv. *trifolii*, protein structural comparisons between these additional NodD regulators and the flavonoid–NodD1 demonstrate low conservation in the predicted flavonoid binding sites. These data suggest that an alternative molecule could act as a NodD protein inducer, particularly since these specific NodD proteins are active when rhizobia are inside the roots or nodules compared with the rhizosphere.

The ability to recognize different flavonoids is the primary rhizobial determinant of host range (Walker *et al.*, 2020). Therefore, the presence of more than one *nodD* in the genome is generally associated with a broader host-range ability. *Rhizobium tropici* CIAT 899 (hereafter CIAT 899) is a broad host range rhizobial strain that nodulates some legumes, including *Phaseolus vulgaris*, *Lotus japonicus*, and *Lotus burttii* (Martínez-Romero *et al.*, 1991; del Cerro *et al.*, 2015a, b). Genome sequencing of CIAT 899 revealed five different *nodD* genes located in the symbiotic plasmid (Ormeño-Orrillo *et al.*, 2012). Interestingly, the CIAT 899 *nodD2* gene is up-regulated and active in the presence of osmotic stress, resulting in the activation of *nod* gene expression even in the absence of flavonoids, raising hypotheses that an osmotic stress-dependent molecule could act as a NodD2 inducer or that the increase in *nodD2* expression alone could be responsible for NF production (Pérez-Montaño *et al.*, 2016; del Cerro *et al.*, 2017, 2019). A *nodD2* mutant exhibited detrimental effects on the symbiotic performance in its natural host, *Phaseolus vulgaris*, but symbiotic ability in the other host plants, *Lotus japonicus* and *Lotus burttii*, was unaffected (del Cerro *et al.*, 2017), which could be due to genetic *nodD* redundancy. Among additional NodD proteins, NodD1 is active in the presence of compatible flavonoids and its absence abolishes nodulation in *L. japonicus* and reduces nodulation in *P*. *vulgaris* and *L*. *burttii* (Ormeño-Orrillo *et al.*, 2012; del Cerro *et al.*, 2015a, b). However, a double mutant in *nodD1* and *nodD2* genes abolishes nodulation in all three plant species confirming the relevance of the flavonoid-activated NodD1 and osmotic stress-activated NodD2 proteins during symbiosis.

Here, we demonstrate that NodD2 does not require activation by inducers such as compatible flavonoids or an osmotic stress-dependent molecule. We show that overexpression of *nodD2* alone results in a strong induction of *nod* genes in any tested condition. Furthermore, we demonstrate that a strain overexpressing *nodD2*—but lacking all additional *nodD* genes including *nodD1*—can nodulate *P*. *vulgaris*, *L. japonicus* and *L. burttii* as efficiently as the wild-type (WT) strain. Finally, we show that the overexpression of *nodD2* in *Sinorhizobium fredii* HH103 results in nodule development in the incompatible non-host *P. vulgaris* and increased the number of nitrogen-fixing nodules in hosts such as *L. japonicus* and *L. burttii*. The finding that overexpression of *nodD2* alone can increase host range and symbiotic efficiency in several legume plants could lead to exciting opportunities for developing novel rhizobial inoculants to increase crop production.

## Material and methods

### Bacterial growth conditions, plasmids, and obtaining mutant

All strains and plasmids used in this work are listed in Supplementary Table S1. *Escherichia coli* strains were cultured on LB medium (Sambrook *et al.*, 1989) at 37 °C. *Rhizobium tropici* CIAT 899 strains were grown at 28 °C on tryptone yeast (TY) medium (Beringer, 1974), B^−^ minimal medium (Spaink *et al.*, 1992), or modified yeast extract mannitol (YM3, with 3 g ml^−1^ of mannitol) medium (Vincent, 1970), supplemented, when necessary, with 3.7 μM apigenin, 300 mM NaCl, or 400 mM mannitol.

The quintuple ∆*nodD1–5* deletion mutant was generated using five rounds of overlapping PCR extensions: the double *∆nodD1/∆nodD2* mutant (del Cerro *et al.*, 2017) was used as a recipient to generate Δ*nodD123*, which was subsequently used to obtain a quadruple mutant, the base for the construction of the quintuple ∆*nodD12345* mutant. In total, 919, 328, 756, 930 and 930 bp lengths were deleted from *nodD1* (RTCIAT899_PB01295), *nodD2* (RTCIAT899_PB01070), *nodD3* (RTCIAT899_PB00640), *nodD4* (RTCIAT899_PB01560), and *nodD5* (RTCIAT899_PB00560), respectively. The deleted *nodD3–5* genes were first subcloned into the pK18*mobsacB* vector generating the pK18*mobsacB*::∆*nodD3*, pK18*mobsacB*::∆*nodD4*, and pK18*mobsacB*::∆*nodD5* vectors. Then, plasmids were transferred by conjugation and integrated into the genome of the corresponding strain by single recombination. A double homologous recombination event, in which the WT copy of the gene together with plasmid pK18mob*sacB* were lost, was selected for each *nodD* gene. Mutated strains were confirmed by PCR and DNA–DNA hybridization. For this, DNA was blotted to Hybond-N nylon membranes (Sigma), and the DigDNA method of Roche (Basel, Switzerland) was employed according to the manufacturer’s instructions. The *in cis* complementation of the different *nodD* genes (LE *nodD* strains) was performed by integration of the different pK18mob*sacB*::*nodD* plasmids into the genome of the ∆*nodD1–5* mutant, obtaining derivative strains that harbour both WT and deleted versions of the corresponding *nodD* gene. Complementation *in trans* (OE *nodD* strains) *nodD* versions was carried out by cloning full open reading frame and own promoter sequences into the multicopy vector pBBR-1-MCS-5. The resulting plasmids were individually introduced in ∆*nodD1–5* by conjugation, obtaining derivative strains that constitutively overexpress single *nodD* genes. Complemented strains were confirmed by PCR. Plasmid pMP220::P_nodA1_, obtained as in del Cerro *et al.* (2020), bearing the promoter region of the *nodA1* gene upstream of the *lacZ* reporter gene was conjugated into the different CIAT 899 WT and derivative strains. Plasmid pFAJDsRed, which harbours a gene for red fluorescence, was also introduced by conjugation in CIAT 899 and derivatives (Kelly *et al.*, 2013). All plasmids were transferred from *Escherichia coli* to CIAT 899 by conjugation as previously described using plasmid pRK2013 as the helper (Simon, 1984). Recombinant DNA techniques were performed according to the general protocols of Sambrook *et al.* (1989). Primers used in this study are listed in Supplementary Table S2.

### RNA extraction, cDNA synthesis, and quantitative RT-PCR

RNA extraction, cDNA synthesis and quantitative RT-PCR (qPCR) experiments were performed as previously described (Pérez-Montaño *et al.*, 2016). Briefly, total RNA was isolated from bacterial cultures grown in TY medium using a High Pure RNA Isolation Kit (Roche) and RNAase Free DNA Set (Qiagen, USA), according to the manufacturer’s instructions. Verification of the amount and quality of RNA samples was carried out using a Nanodrop 1000 spectrophotometer (Thermo Fisher Scientific, USA) and a Qubit 2.0 Fluorometer (Thermo Fisher Scientific). Two independent total RNA extractions were obtained for each condition.

This (DNA-free) RNA was reverse transcribed into cDNA using PrimeScript RT reagent Kit with gDNA Eraser (Takara, Japan). Quantitative PCR was performed using a LightCycler 480 (Roche) with the following conditions: 95 °C, 10 min; 95 °C, 30 s; 55 °C, 30 s; 72 °C, 20 s; 40 cycles, followed by the melting curve profile from 60 to 95 °C to verify the specificity of the reaction. The *R. tropici* CIAT 899 16S rRNA gene was used as an internal control to normalize gene expression. The fold-changes of two biological samples with three technical replicates of each condition were obtained using the ΔΔ*C*_t_ method (Pfaffl, 2001). Selected genes and primers are listed in Supplementary Table S2.

### Determination of β-galactosidase activity

To determine β-galactosidase activity, complemented strains were conjugated with the plasmid pMP220::P_nodA1_ (del Cerro *et al.*, 2020) containing a transcriptional fusion between the CIAT 899 *nodA1* promoter and the *lacZ* gene. Assays of β-galactosidase activity were carried out as described by Zaat *et al.* (1987) from bacterial cultures grown in YM3 medium. Units of β-galactosidase activity were calculated according to Miller (1972). The experiments were repeated three times.

### Identification of NF

Chemical identification of the NF was carried out with a ultra-high-pressure liquid chromatography system coupled to a mass spectrometer (UHPLC-MS/MS) as previously described by Jiménez-Guerrero *et al.* (2020a), growing the WT and the derivative mutant strains in B^−^ minimal medium. The NF detected that presented lower area values than 10^7^ in the UHPLC-MS/MS analyses were discarded.

### Protein alignment and secondary structure modelling

The protein sequence alignment of the five NodD proteins was performed with PROMALS3D (Pei *et al.*, 2008) and displayed with Jalview software using the homology colouring scheme (Waterhouse *et al.*, 2009). A percentage identity matrix was created by Clustal 2.1. Models for the secondary structure of NodD1 and NodD2 were made with Phyre2 (Kelley *et al.*, 2015), a web server for protein modelling that uses homology detection methods to build three-dimensional models of proteins, using as a reference the predicted structure of NodD1 from *Sinorhizobium meliloti* (Peck *et al.*, 2013).

### Nodulation assays

For the evaluation of the symbiotic phenotypes, the different CIAT 899 strains were grown in YM3 medium until the bacterial concentration reached about 10^9^ cells ml^−1^. Surface-sterilized seeds of *P. vulgaris*, *L. burttii*, and *L. japonicus* were pre-germinated and placed on sterilized jars (for *P*. *vulgaris*, Leonard jars) containing vermiculite and perlite substrates (3:1) and Farhaeus N-free solution as previously described (del Cerro *et al.*, 2015a, b). Each pre-germinated and sterilized seed was inoculated with 1 ml of bacterial culture. Growth conditions were 16 h at 26 °C in the light and 8 h and 18 °C in the dark, with 70% of humidity. Nodulation parameters were evaluated after 30 d for *P. vulgaris* or 50 d for *Lotus* plants. Nodulation experiments were performed two times.

### Microscopy

*Lotus burttii* and *L. japonicus* plants were grown on ¼ B&D medium (Kelly *et al.*, 2018) in 12 cm square plates at 21 °C with 16 h–8 h day–night cycles. For observing nodules, 21-day-old *Lotus* nodules induced by the different strains carrying the DsRed fluorescent marker were embedded in 6% agarose in water and sliced in thick layer sections (30–50 μm) using a Leica VT 1000S vibratome. Sections of nodules were stained with 0.04% calcofluor (Kawaharada *et al.*, 2017) and observed using a Zeiss LSM510 META confocal microscope. For observing infection threads (ITs), *L. burttii* and *L. japonicus* seedlings grown in square plastic dishes were inoculated with the different strains carrying the DsRed fluorescent marker, 7 d after germination. ITs were observed by Zeiss Axioplan 2 imaging using an Olympus BX53 microscope (Olympus, Japan). Image pictures were merged by using CellSens standard software (Olympus).

## Results

### NodD2 strongly induces the expression of the *R*. *tropici* CIAT 899 *nod* genes and NF synthesis in the absence of inducers

*Rhizobium tropici* CIAT 899 has five NodD proteins. NodD1 and NodD2 are the main *nod*-gene regulators since a double *nodD1/nodD2* mutant abolishes NF production and impairs nodule formation in *P*. *vulgaris* and *L*. *burttii* (del Cerro *et al.*, 2017). It is unknown whether each NodD protein can function alone because the function of each of the five individual NodD proteins—without additional NodD proteins in the background—was not characterized and genetic redundancy among the five *nodD*-gene copies could mask potential phenotypes. To better understand the role of the five NodD proteins individually, we expressed them in single- or multi-copy vectors in a strain lacking any *nodD* gene in a quintuple *ΔnodD1–5* mutant. First, we mutagenized by gene-deletion the *nodD3*, *nodD4*, and *nodD5* genes of CIAT 899 in the double *∆nodD1/∆nodD2* mutant strain from our previous study (del Cerro *et al.*, 2017) and obtained a quintuple *∆nodD1*–*5* deletion mutant strain. We confirmed the deletion of the five *nodD* copies by DNA–DNA hybridization and PCR (Supplementary Fig. S1A). Then, we subcloned each *nodD* sequence preceded by its own promoter region into single- and multi-copy vectors. We used pK18mob*sacB* for the single-copy vector because this non-replicable vector is recombined into the genome and pBBR-1-MCS-5 for the multi-copy vector since it does not recombine into the genome and it replicates in rhizobia. We introduced these vectors independently into the quintuple *∆nodD1*–*5* mutant strain by conjugation. Thus, we generated strains that expressed each *nodD* gene at either low or high levels (Supplementary Table S1). We quantified their respective expression in each strain via qRT-PCR confirming the low or high expression of each *nodD* gene (Supplementary Fig. S1B). Consequently, we named these new strains according to their *nodD* relative expression: *nodD*-LE (low-expression, single-copy vector) and *nodD*-OE (overexpression, multi-copy vector).

**Fig. 1.**
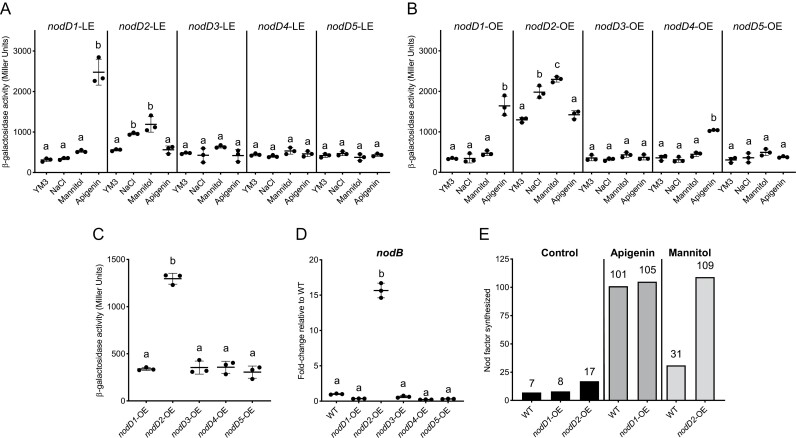
*Rhizobium tropici* CIAT 899 NodD2 induces the expression of *nod* genes in the absence of inducers. *Rhizobium tropici* CIAT 899 quintuple *nodD* mutant strains carrying CIAT 899 *nodA1* promoter fused to the *lacZ* gene and expressing *nodD* genes individually at low (LE), or high (overexpression) (OE) levels. (A, B) β-Galactosidase activity of the LE (A) and OE (B) strains during different treatments: control (YM3), flavonoid treatment (YM3 + 3.7 µM flavonoid apigenin), and osmotic stress treatments (YM3 + 300 mM NaCl, or YM3 + 400 mM mannitol). (C) β-Galactosidase activity of the OE strains during control (YM3) treatment. (D) Gene expression of *R. tropici* CIAT 899 *nodB* during control (YM3) treatment in WT and *nodD*-OE strains. Expression levels were measured by qRT-PCR, normalized to 16S rRNA gene, and plotted relative to WT. (E) Number of NFs produced by WT, *nodD1*-OE, and *nodD2*-OE in the presence and the absence of inducers measured by UHPLC-MS/MS. Plots (C, D) represent the means (lines), standard deviation (error bars), and individual biological replicates (dots). In (A–D) a one-way ANOVA was used to test for differences between mutants with a Tukey correction for multiple comparisons (same letters represent no significant difference at 0.05% level).

Previous studies show that NodD1 and NodD2 induce *nod-*gene expression in the presence of flavonoids and osmotic stress, respectively (Pérez-Montaño *et al.*, 2016; del Cerro *et al.*, 2017, 2019). Thus, we investigated *nod*-gene expression in our strains under different conditions: control (YM3 medium), flavonoid treatment (YM3 medium + the flavonoid apigenin), and osmotic stress treatments (YM3 medium + salt, or YM3 medium + mannitol). To explore this, we transferred the pMP220 vector containing the promoter of the main operon of CIAT 899 *nod* genes fused to the *lacZ* reporter gene (P_nodA1_::*lacZ*) into our strains and performed β-galactosidase activity assays to determine transcriptional activation of these *nod* genes. The *nodD1-*LE and *nodD1-*OE exhibited increased β-galactosidase activity with the flavonoid apigenin treatment whereas *nodD2-*LE and *nodD2-*OE exhibited increased β-galactosidase activity with both osmotic stress treatments (salt and mannitol) (Fig. 1A, B). The other strains, *nodD3-*LE, *nodD3-*OE, *nodD4-*LE, *nodD4-*OE, *nodD5-*LE, and *nodD5-*OE, did not show enhanced β-galactosidase activity with any treatment, except *nodD4-*OE, which presented higher activity with the flavonoid apigenin. These data confirm that the expression of *nodD1* or *nodD2* alone is sufficient for *nod*-gene expression during flavonoid treatment or osmotic stress treatment, respectively.

Excitingly, we noticed that the *nodD2*-OE strain presented high β-galactosidase activity in the absence of inducers (control treatments) (Fig. 1C). To confirm this result, we used qRT-PCR to quantify the expression of a *nodD*-dependent gene, *nodB*, in the *nodD-*OE strains. In the absence of inducers, *nodB* expression was significantly greater in *nodD2-*OE (over 15-fold) compared with the rest of the strains (Fig. 1D). In summary, these data show that high levels of NodD2 can constitutively activate the transcription of the *nod* genes in the absence of inducers.

We then investigated whether *nod*-gene induction in *nodD2*-OE is correlated with an increase in the number of NFs produced. UHPLC-MS/MS detected a greater number of NFs produced by *nodD2-*OE in the absence of inducers (17), compared with the WT (seven) and *nodD1-*OE (eight) used as control strains (Fig. 1E; Supplementary Dataset S1). When bacterial cultures were supplemented with inducers, up to 101 and 31 NFs were detected in the WT strain in the presence of apigenin and mannitol, respectively. The *nodD1-*OE strain produced a similar number of NFs (105) to the WT strain. However, the number of NFs produced by the *nodD2-*OE strain in the presence of mannitol (109) far exceeded the NFs obtained by the WT strain (31). In summary, our results show that overexpression of the *nodD2* gene alone is both sufficient to activate the induction of the *nod* genes and NF synthesis in the absence of NodD inducers, and results in an increase in the production of NFs in the presence of osmotic stress.

### *NodD2* overexpression allows efficient nodulation in the absence of the other NodD proteins

**Fig. 2.**
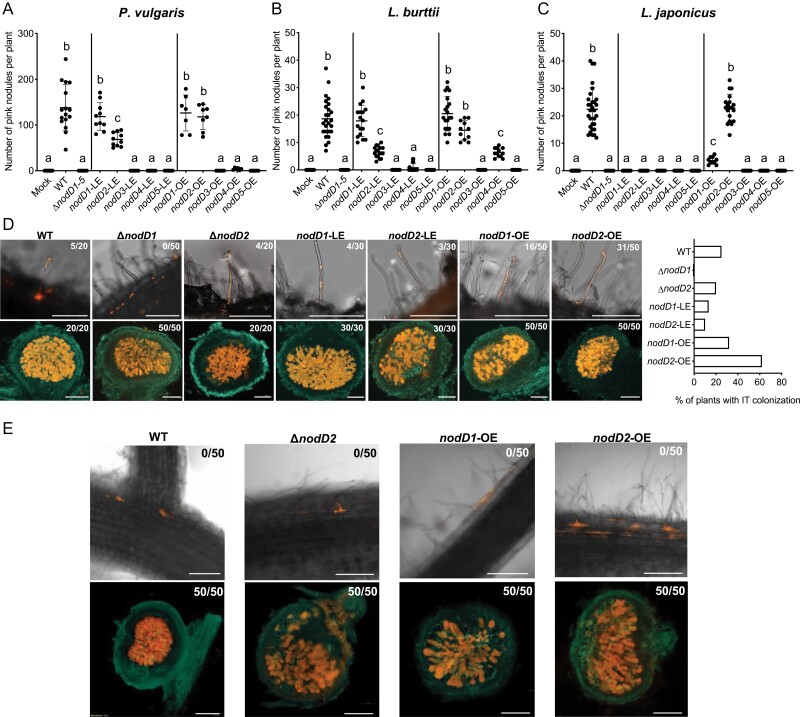
Overexpression of *Rhizobium tropici* CIAT 899 *nodD2* in a quintuple *nodD* mutant restores nodulation in *Phaseolus vulgaris*, *Lotus japonicus*, and *Lotus burttii*, and enhances the incidence of infection thread formation in *L. burttii.* (A–C) Number of pink (nitrogen-fixing nodules) in *P. vulgaris* (A, B), and *L. japonicus* (C) plants quantified 30 (A) or 50 (B, C) days post-inoculation with *R. tropici* CIAT 899; quintuple *nodD* mutant (*∆nodD1–5*); and *nodD* quintuple mutant strains expressing *nodD* genes individually at low levels (LE), or high (overexpression) levels (OE). Plots represent the means (lines), standard deviation (error bars), and individual biological replicates (dots). A one-way ANOVA was used to test for differences between mutants with a Tukey correction for multiple comparisons (same letters represent no significant difference at 0.05% level). (D) Infection threads (top panel) and fully colonized nodules (lower panel) of *L. burttii* plants inoculated with *R. tropici* CIAT 899 WT, single deletion mutants *∆nodD1* and *∆nodD2*, and quintuple deletion mutant strains expressing *nodD1* or *nodD2* (*nodD1*-LE, *nodD2*-LE, *nodD1*-OE, and *nodD2*-OE) harbouring *DsRed* fluorescent marker. The number of plants with infection threads or nodules is indicated on the top right-hand side of each image. Scale bar: 200 μm. (E) Intercellular entry (top panel) and fully colonized nodules (lower panel) of *L. japonicus* plants inoculated with *R. tropici* CIAT 899, single deletion mutant *∆nodD2*, and quintuple deletion mutant strains over-expressing *nodD1* or *nodD2* (*nodD1*-OE and *nodD2*-OE) harbouring *DsRed* fluorescent marker. The number of plants with infection threads or nodules is indicated on the top right-hand side of each image. Scale bar: 200 μm.

CIAT 899 establishes symbiotic interactions with a wide range of hosts from the three legume subfamilies (Hungria *et al.*, 2000). In previous studies, we determined that NodD1 is essential for nodulation in *L*. *japonicus*, whereas both NodD1 and NodD2 are involved in nodulation in *P*. *vulgaris* and *L*. *burttii* (del Cerro *et al.*, 2017). To better understand the individual role of the different NodD proteins in the symbiosis with these plant species, we studied nodulation of the LE and OE *nodD* strains in *P*. *vulgaris*, *L*. *burttii*, and *L. japonicus*. As expected, the *∆nodD1–5* strain did not form nodules in these legumes (Fig. 2A–C). In *P. vulgaris* and *L. burttii*, *nodD1-*LE, *nodD1-*OE, and *nodD2-*OE formed pink nitrogen-fixing nodules at similar levels to the WT strain (Fig. 2A, B). The *nodD2-*LE strain also restored the formation of pink nitrogen-fixing nodules, but at lower levels than the WT strain. Excitingly, in *L*. *japonicus*, only the *nodD2-*OE strain formed nodules in similar numbers to the WT strain, while *nodD1-*OE formed fewer pink nodules (Fig. 2C). None of the LE strains formed nodules in *L*. *japonicus*—except *nodD1*-LE which formed white nodules—indicating that the other *nodD* copies might be required for root/nodule colonization in this plant (Supplementary Fig. S2). To determine whether nodulation efficiency was correlated to improved plant performance, we quantified shoot dry weight (SDW) in plants inoculated with the *nodD-*LE and *nodD-*OE strains (Supplementary Fig. S3A, B). The plants inoculated with the quintuple deletion mutant exhibited reduced SDW compared with those inoculated with the WT strain due to the lack of rhizobial nitrogen fixation events. In *P. vulgaris* and *L. burttii*, the SDW of the plants inoculated with *nodD2*-LE, *nodD1-*OE, or *nodD2-*OE was similar to those inoculated with the WT strain, whereas the plants inoculated with *nodD1*-LE showed lower SDW in *L*. *burttii*. In *L*. *japonicus*, none of the plants inoculated with the LE and OE reached the SDW levels of the WT. Only the plants inoculated with *nodD1*-OE and *nodD2*-OE strains presented higher SDW than the *∆nodD1–5* mutant. The three legumes inoculated with the additional *nodD-*LE or *nodD-*OE strains showed similar SDW to the plants inoculated with the *∆nodD1–5* mutant. In summary, these results suggest that in the absence of other NodD proteins (including NodD1), a low expression of *nodD2* alone (*nodD2-*LE) is sufficient for forming nitrogen-fixing nodules in *P*. *vulgaris* and *L*. *burttii*. Indeed, in all three plant species, overexpression of *nodD2* alone is sufficient to completely restore nodulation to WT levels.

### CIAT 899 mainly infects *L. burttii* plants intercellularly but the overexpression of *nodD2* enhances infection thread formation

CIAT 899 colonizes its natural host (*P*. *vulgaris*) via the formation of infection threads (IT) in root hairs, in a process dependent on NF (Rodríguez-López *et al.*, 2019). In *L. burttii* and *L. japonicus*, both NodD1 and NodD2 are involved in nodulation (Fig. 2B, C). However, to this date, the infection mechanism in *Lotus* by CIAT 899 (IT formation *versus* intercellular entry) and the role of the NodD proteins in the infection process remain unknown.

To establish how CIAT 899 colonizes *L*. *burttii* and *L. japonicus*, we examined IT formation and nodule occupancy in both plants inoculated with CIAT 899. We observed low numbers of ITs in only 5/20 plants (25%) but fully colonized root and nodules in all plants (20/20), which points to the fact that CIAT 899 mainly colonizes *L*. *burttii* intercellularly (Fig. 2D; Supplementary Fig. S4). In the case of *L. japonicus*, no ITs were observed in any plant inoculated with CIAT 899 but fully colonized roots and nodules were observed in all analysed plants (50/50), indicating that CIAT 899 colonizes *L*. *japonicus* intercellularly (Fig. 2E). To investigate the individual contribution of NodD1 and NodD2 to the low incidences of IT formation in *L. burttii*, we examined the presence of ITs in plants inoculated with two single deletion mutants (*∆nodD1* and *∆nodD2*) obtained in a previous study (del Cerro *et al.*, 2017). We observed no ITs in plants inoculated with the *∆nodD1* mutant; however, fully colonized roots and nodules were formed (Fig. 2D). These data suggest that NodD1 is required for IT formation in *L. burttii*. After inoculation with the *∆nodD2* mutant, the proportion of plants presenting ITs (4/20) was similar to the WT strain (5/20). These results suggest that NodD2 is not essential for IT formation. To confirm whether NodD2 alone contributes to IT formation, we quantified IT numbers in the plants inoculated with *nodD2-*LE and found that 3/30 plants presented IT formation, a similar level to the plants inoculated with the *nodD1*-LE strain (4/30), suggesting that NodD2 also has a role in IT intracellular colonization.

We then investigated if the overexpression of NodD1 or NodD2 increases incidences of IT-type colonization. We found that *nodD1-*OE slightly increased the proportion of ITs compared with the WT (32% versus 25%, respectively). Excitingly, the plants inoculated with the *nodD2*-OE largely increased the proportion of ITs (62%). In summary, these data suggest that NodD2 has a role in IT formation and the overexpression of NodD2 increases IT-type colonization.

### Nodule formation and colonization can be extended to other rhizobial strains by overexpression of NodD2 of CIAT 899

*Sinorhizobium fredii* HH103 (hereafter HH103)—a phylogenetically distant rhizobial strain from CIAT 899—cannot form nodules in *P*. *vulgaris* but forms white (non-colonized) nodules in *L*. *japonicus* and nitrogen-fixing nodules in *L*. *burttii* by intercellular infection (Fuentes-Romero *et al.*, 2022). Interestingly, HH103 derivative strains overproducing NF gain the capacity for IT colonization in both *Lotus* plants and, most importantly, fully colonize nodules in *L. japonicus* (Acosta-Jurado *et al.*, 2019).

**Fig. 3.**
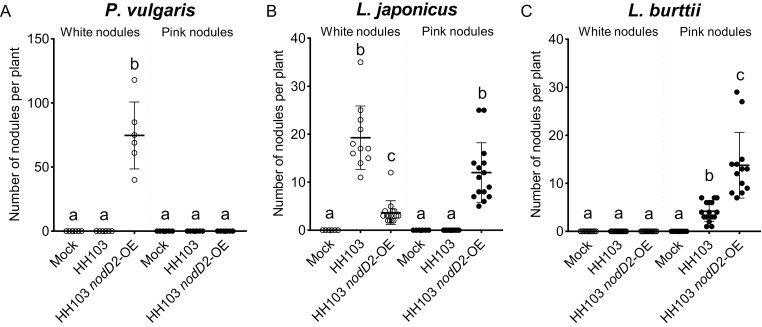
Overexpression of *Rhizobium tropici* CIAT 899 *nodD2* extends the host range of *Sinorhizobium fredii* HH103. Numbers of white (non-colonized) and pink (nitrogen-fixing) nodules in *P. vulgaris* (A), *L. burttii* (B), and *L. japonicus* (C) plants quantified 30 (A) or 50 (B, C) days post-inoculation with *Sinorhizobium fredii* HH103 strain containing the *nodD2*-OE vector. Plots represent the means (lines), standard deviation (error bars), and individual biological replicates (dots). A one-way ANOVA was used to test for differences between mutants with a Tukey correction for multiple comparisons (same letters represent no significant difference at 0.05% level).

To determine whether CIAT 899 NodD2 can also extend symbiotic ability in another rhizobial species, we introduced the *nodD2-*OE vector into HH103. Excitingly, HH103 *nodD2-*OE formed white (non-colonized) nodules in *P. vulgaris*, whereas the WT stain could not (Fig. 3A). This result suggests that the CIAT 899 constitutively active NodD2 protein can extend the host range of HH103. In *L*. *japonicus*, HH103 formed white nodules, but the HH103 *nodD2*-OE formed both white and pink nodules (Fig. 3B). This result shows that NodD2 can promote root and nodule colonization even in a different rhizobial strain. Finally, in *L. burttii*, HH103 *nodD2*-OE formed more effective (pink) nodules than WT HH103 (Fig. 3C). These results indicate that NodD enhances symbiotic efficiency in *S. fredii* HH103.

## Discussion

The success of the rhizobium–legume symbiotic interaction, including root infection, nodule formation and invasion, depends on the ability of the bacteria to recognize specific flavonoids released by the roots. NodD proteins are important determinants of host-range specificity: they bind only to compatible flavonoids to initiate the transcription of the *nod* genes and subsequent progression of the symbiotic process (Horvath *et al.*, 1987; Spaink *et al.*, 1989). Increasing compatibility between rhizobia and legume hosts could benefit sustainable agriculture (Jiménez-Guerrero *et al.*, 2020b). Thus, different studies have aimed to reduce the specificity of this symbiotic interaction; for example, specific mutations or amino acid substitutions in the NodD protein exhibit a flavonoid-independent transcriptional activation (FITA mutants) of the *nod* genes and therefore NF synthesis (McIver *et al.*, 1989; Vinardell *et al.*, 2004; Hou *et al.*, 2009; Peck *et al.*, 2013). A recent study suggested that rhizobia could harbour naturally active NodD proteins as some species of *Rhizobium leguminosarum* sv. *trifolii* presented a NodD2 copy which may induce the *nod* genes without flavonoid inducers (Ferguson *et al.*, 2020). Here, we have demonstrated by β-galactosidase assays and qRT-PCR that *R*. *tropici* CIAT 899 NodD2 alone can induce the *nod* genes in the absence of inducers (Fig. 1). In our previous studies, we found that *nodD2* expression was induced by osmotic stress (salt or mannitol) but not by the flavonoid apigenin and we demonstrated that the activation of downstream *nod* genes under these conditions was NodD2-dependent (Pérez-Montaño *et al.*, 2016; del Cerro *et al.*, 2017, 2019).

Here, our results suggest that NodD2 is naturally active, i.e. does not require activation by inducers such as compatible flavonoids or an osmotic stress-dependent molecule. Thus, the NodD2-dependent activation of *nod* genes observed in our previous studies results from increased *nodD2* expression under osmotic stress but not from the presence of inducers *per se*. In line with these findings, protein alignment of the five CIAT 899 NodD proteins suggests that several regions associated with the predicted flavonoid binding domains of NodD2 have reduced conservation, despite the high overall homology observed in the alignment (Supplementary Fig. S5). Thus, pairwise comparisons between NodD1, NodD3, NodD4, and NodD5 show amino acid sequence similarities between 67.08% and 77.78%, whereas pairwise comparisons between NodD2 and the other NodD proteins ranged lower, between 54.81% and 61.38 % (Supplementary Fig. S5A). Since NodD1 is activated by compatible flavonoids but NodD2 is constitutively active (does not require inducers), we searched for differences in the predicted ligand-binding domain between these proteins, which correspond to three domains in positions L^133^–R^143^, D^154^–R^163^ and A^198^–I^208^ in NodD1 of CIAT 899 (Supplementary Fig. S5B). Here, the predicted ligand-binding domains of NodD2 showed less conservation than the other CIAT 899 NodD proteins. Interestingly, *R*. *tropici* CIAT 899 NodD1 has a positively charged arginine, and NodD2 has a polar-charged glutamine in the same position (205) within the A^198^–I^208^ predicted ligand-binding domain. In fact, a substitution mutation in the predicted flavonoid binding domain of NodD1 in *Sinorhizobium meliloti*, a positively charged lysine to a polar uncharged asparagine (K^205^N), resulted in constitutive activation of the protein in the absence of flavonoids (Peck *et al.*, 2013). Altogether, these differences in sequence and predicted structure could explain the different activation patterns observed between NodD1 and NodD2 of CIAT 899.

We hypothesize that these differences may explain the different activation requirements observed between NodD1 and NodD2 of CIAT 899. This is also in agreement with previous studies that predict that disparate inducers activate NodD1 and NodD2 proteins of *Mesorhizobium loti* R7A or *R. leguminosarum* bv. *trifolii* TAI (Kelly *et al.*, 2018; Ferguson *et al.*, 2020). However, despite decades of research, the structure of NodD proteins remains elusive, mostly because NodD could not be purified alone (Fisher *et al.*, 1988; Yeh *et al.*, 2002; Kostiuk *et al.*, 2013). The up-regulation of *nod* genes via NodD2 is likely to require additional regulators, since the presence of an AraC family regulator (OnfD) that interacts with NodD2 is also essential for the induction of the *nod* genes under osmotic stresses in CIAT 899 (del Cerro *et al.*, 2020). Interestingly, in *S. fredii* HH103, the protein encoded by SFHH103_06433 possesses similarity (~64%) to OnfD, which could be assisting NF production in this rhizobial strain when the CIAT 899 NodD2 regulator is introduced.

A remaining question arising from these findings is whether the NFs produced upon NodD2 activation are symbiotically active. Our results indicate that low expression *nodD2* alone is sufficient to partially restore nodulation in *P. vulgaris* and *L. burttii* and restored SDW in both plants to WT levels (Fig. 2A, B; Supplementary Fig. S3). These data suggest that *nodD2* is important for symbiotic efficiency, especially during the later stages of nodulation. Previous studies pointed out different roles of NodD1 and NodD2 of *M*. *loti* R7A, and NodD2 of *R. leguminosarum* bv. *trifolii* in root/nodule colonization (Kelly *et al.*, 2018; Ferguson *et al.*, 2020). In our study, the overexpression of *nodD2* seems to have an impact at all infection stages: in the rhizosphere before infection, inside root hairs, and in nodules. In fact, we showed that the overexpression of NodD2 alone fully complemented nodulation in all three legumes assayed and promoted IT formation in *L. burttii* (Fig. 2A–D). Furthermore, overexpression of CIAT 899 NodD2 in *S. fredii* HH103 promoted initial nodule formation in a naturally incompatible host and enabled the formation of nitrogen-fixing nodules in a host whose infection process cannot progress to later colonization stages (*L. japonicus*) (Fig. 3A, B). These results highlight the ability of NodD2 to function in multiple stages of the rhizobial interaction.

To this date, two different modes of root colonization by rhizobia have been reported. The best studied is the formation of ITs in the root hairs that progress through the cortical cells to reach the nodule primordia in a process dependent on NFs. This type of root infection is associated with high compatibility rhizobium–legume interactions and can be observed in the model legumes *L*. *japonicus* and *M*. *truncatula*, in addition to the crop legumes such as *Glycine max* (soybean) and *P*. *vulgaris* (common bean) (Acosta-Jurado *et al.*, 2019; Rodríguez-López *et al.*, 2019; Montiel *et al.*, 2021). The other mode of infection—known as intercellular entry—represents a less evolved and less sophisticated mechanism, which consists of root invasion between epidermal cells or through cracks generated by the growth of lateral roots (González-Sama *et al.*, 2004; Goormachtig *et al.*, 2004; Sprent, 2007). *Rhizobium tropici* CIAT 899 is generally associated with an IT mode of infection in its natural host, *P*. *vulgaris* (Martínez-Romero *et al.*, 1991; Rodríguez-López *et al.*, 2019). Despite this evolved way of invasion being also detected with its less compatible host *L*. *burttii*, here we show that this bacterium mainly colonizes this plant by an intercellular mechanism (Fig. 2D; Supplementary Fig. S4). In *L*. *burttii*, both types of root infection have been observed: different strains of *M*. *loti* present IT formation, whereas *Sinorhizobium fredii* HH103 shows intercellular infection (Kelly *et al.*, 2018; Acosta-Jurado *et al.*, 2016; Jiménez-Guerrero *et al.*, 2020a). Thus, it is not surprising that CIAT 899 can colonize this plant via ITs and intercellularly, which is an ideal scenario for studying whether the NodD proteins are involved in the interplay of both types of root colonization. Our results indicate that the CIAT 899 NodD1 is essential for the formation of ITs, whereas NodD2 can enhance this mode of infection since overexpression of the CIAT 899 NodD2 increases the percentage of plants presenting ITs. In summary, all these results demonstrate the importance of NodD2 for the success of *R. tropici* CIAT 899 in the symbiotic process.

Previous studies did not identify a specific role for additional NodD proteins (NodD3, NodD4, and NodD5) since single mutants do not exhibit large differences in nodulation with these legumes (del Cerro *et al.*, 2015a). In this study, we bypassed the putative genetic redundancy of the other *nodD* genes in the single mutants. The overexpression of *nodD4* in the quintuple *nodD* mutant resulted in a small increase in expression of *nod* genes (quantified via β-galactosidase assay) when treated with the flavonoid apigenin and led to the partial complementation of nodule number in *L*. *burttii* (Figs 1B, 2B). These results indicate that NodD4 can recognize flavonoids and induce *nod* gene expression in the absence of the other NodD proteins.

NodD proteins are responsible for the specificity between host plants and rhizobia. However, compatibility is often limited by the ability to recognize specific flavonoids (Walker *et al.*, 2020). In this work, we show that NodD2 bypasses this requirement because it can constitutively activate *nod* gene transcription: this presents opportunities for modifying rhizobial host range in a flavonoid-independent manner. Indeed, orthologous NodD proteins can function in disparate rhizobial species (Fuentes-Romero *et al.*, 2022), and here we demonstrate that the overexpression of *nodD2* in *S. fredii* HH103 results in the nodule formation of a normally incompatible non-host *P. vulgaris*, and increases colonization in hosts *L. japonicus* and *L. burttii* (Fig. 3). This finding has considerable agricultural importance because increasing the compatibility between rhizobia and plants could be potentially used to develop novel rhizobial inoculants for crops.

## Supplementary data

The following supplementary data are available at *JXB* online.

Fig. S1. Relative gene expression of *Rhizobium tropici* CIAT 899 *nodD1*, *nodD2*, *nodD3*, *nodD4*, and *nodD5* in a quintuple deletion mutant strain expressing the five *nodD* genes individually.

Fig. S2. Number of white (non-colonized) nodules in *L. japonicus* plants quantified 50 d post-inoculation with *Rhizobium tropici* CIAT 899 strains.

Fig. S3. Shoot dry weights in *Phaseolus vulgaris*, *Lotus japonicus*, and *Lotus burttii* plants inoculated with *Rhizobium tropici* CIAT 899 strains.

Fig. S4. Infection threads of *Lotus burttii* and *Lotus japonicus* plants inoculated with *Mesorhizobium loti* R7A and intercellular entry of *L. burttii* plants inoculated with *R. tropici* CIAT 899 strains.

Fig. S5. Amino acid alignments of *Rhizobium tropici* CIAT 899 NodD1, NodD2, NodD3, NodD4, and NodD5 proteins.

Table S1. Bacterial strains and plasmids used in this study.

Table S2. Primers used in this study.

Dataset S1. Nod Factors produced by *Rhizobium tropici* CIAT 899 strains grown in the presence or the absence of *nod* gene inducers.

erac325_suppl_Supplementary_Figures_and_Tables

erac325_suppl_supplementary_Dataset_S1

## Data Availability

All data supporting the findings of this study are available within the paper and within its supplementary materials published online.
