# Supplementary material for: The Rhizobium tropici CIAT 899 NodD2 protein promotes symbiosis and extends rhizobial nodulation range by constitutive nodulation factor synthesis
Source: J Exp Bot. 2022 Jul 28;73(19):6931–41. doi: 10.1093/jxb/erac325 (PMC9629785; doi:10.1093/jxb/erac325)
Supplement: erac325_suppl_Supplementary_Figures_and_Tables [file erac325_suppl_supplementary_figures_and_tables.pdf]

The *Rhizobium tropici* CIAT 899 NodD2 protein promotes symbiosis and extends rhizobial nodulation range by constitutive nodulation factor synthesis.

Running title: The CIAT 899 NodD2 ensures nodulation.

Ayala-García *et al*. 2022

Supplementary files:

- Supplementary Tables 1 and 2.
- Supplementary Figure legends 1, 2, 3, 4 and 5.
- Supplementary Dataset 1.

Table S1. Bacterial strains and plasmids used in this study.

Plasmid or strains	Relevant characteristics	Source of reference
Plasmids		
pBBR1-MCS-5	Multi-copy expression vector (Gm^R)	Kovach *et al.*, 1995
pK18*mobsacB*	Rhizobial suicide vector containing the *sacB* counter-selection gene (Km^R)	Schafer *et al.*, 1994
pMP220	*lacZ* gene without its own promoter (Tc^R)	de Maagd *et al.*, 1988
pFAJDsRed	Constitutively expression of DsRED (red fluorescence) (Tc^R)	Kelly *et al.*, 2013
pRK2013	Conjugation helper vector (Km^R)	Simon *et al.*, 1984
pK18*mobsacB*::Δ*nodD3*	*R. tropici* CIAT 899 deleted *nodD3* and own promoter cloned into pK18mobsacB vector	This study
pK18*mobsacB*::Δ*nodD4*	*R. tropici* CIAT 899 deleted *nodD4* and own promoter cloned into pK18mobsacB vector	This study
pK18*mobsacB*::Δ*nodD5*	*R. tropici* CIAT 899 deleted *nodD5* and own promoter cloned into pK18mobsacB vector	This study
pK18*mobsacB*::*nodD1*	*R. tropici* CIAT 899 full-length *nodD1* and own promoter cloned into pK18mobsacB vector	This study
pK18*mobsacB*::*nodD2*	*R. tropici* CIAT 899 full-length *nodD2* and own promoter cloned into pK18mobsacB vector	This study
pK18*mobsacB*::*nodD3*	*R. tropici* CIAT 899 full-length *nodD3* and own promoter cloned into pK18mobsacB vector	This study
pK18*mobsacB*::*nodD4*	*R. tropici* CIAT 899 full-length *nodD4* and own promoter cloned into pK18mobsacB vector	This study
pK18*mobsacB*::*nodD5*	*R. tropici* CIAT 899 full-length *nodD5* and own promoter cloned into pK18mobsacB vector	This study
pBBR1-MCS-5::*nodD1*	*R. tropici* CIAT 899 full-length *nodD1* and own promoter cloned into pBBR1-MCS-5 vector	This study
pBBR1-MCS-5::*nodD2*	*R. tropici* CIAT 899 full-length *nodD2* and own promoter cloned into pBBR1-MCS-5 vector	This study
pBBR1-MCS-5::*nodD3*	*R. tropici* CIAT 899 full-length *nodD3* and own promoter cloned into pBBR1-MCS-5 vector	This study

pBBR1-MCS-5::*nodD4*	*R. tropici* CIAT 899 full-length *nodD4* and own promoter cloned into pBBR1-MCS-5 vector	This study
pBBR1-MCS-5::*nodD5*	*R. tropici* CIAT 899 full-length *nodD5* and own promoter cloned into pBBR1-MCS-5 vector	This study
pMP220::*pnodA1*	*R. tropici* CIAT 899 *nodA1* promoter (*pnodA1*) cloned into pMP220 vector	del Cerro *et al.*, 2020
Escherichia coli species		
DH5α	*supE44 ΔlacU169 hsdR17 recA1 endA1 gyrA96 thi-1 relA1 (Nal*^{r}*)*	Sambrook *et al.*, 1989
Rhizobium species		
Rhizobium tropici CIAT 899	Wild-type strain (Rif^{R})	Martínez-Romero *et al.*, 1991
Δ*nodD1-5*	CIAT 899 *nodD12345* quintuple deletion mutant	This study
nodD1-LE	Δ*nodD1-5* harbouring the pK18*mobsacB*::*nodD1* vector	This study
nodD2-LE	Δ*nodD1-5* harbouring the pK18*mobsacB*::*nodD2* vector	This study
nodD3-LE	Δ*nodD1-5* harbouring the pK18*mobsacB*::*nodD3* vector	This study
nodD4-LE	Δ*nodD1-5* harbouring the pK18*mobsacB*::*nodD4* vector	This study
nodD5-LE	Δ*nodD1-5* harbouring the pK18*mobsacB*::*nodD5* vector	This study
nodD1-OE	Δ*nodD1-5* harbouring the pBBR1-MCS-5::*nodD1* vector	This study
nodD2-OE	Δ*nodD1-5* harbouring the pBBR1-MCS-5::*nodD2* vector	This study
nodD3-OE	Δ*nodD1-5* harbouring the pBBR1-MCS-5::*nodD3* vector	This study
nodD4-OE	Δ*nodD1-5* harbouring the pBBR1-MCS-5::*nodD4* vector	This study
nodD5-OE	Δ*nodD1-5* harbouring the pBBR1-MCS-5::*nodD5* vector	This study
nodD1-LE pMP220::P_{nodA1}	Δ*nodD1-5* harbouring the pK18*mobsacB*::*nodD1* and pMP220::P_{nodA1} vectors	This study
nodD2-LE pMP220::P_{nodA1}	Δ*nodD1-5* harbouring the pK18*mobsacB*::*nodD2* and pMP220::P_{nodA1} vectors	This study
nodD3-LE pMP220::P_{nodA1}	Δ*nodD1-5* harbouring the pK18*mobsacB*::*nodD3* and pMP220::P_{nodA1} vectors	This study
nodD4-LE pMP220::P_{nodA1}	Δ*nodD1-5* harbouring the pK18*mobsacB*::*nodD4* and pMP220::P_{nodA1} vectors	This study
nodD5-LE pMP220::P_{nodA1}	Δ*nodD1-5* harbouring the pK18*mobsacB*::*nodD5* and pMP220::P_{nodA1} vectors	This study
nodD1-OE pMP220::P_{nodA1}	Δ*nodD1-5* harbouring the pK18*mobsacB*::*nodD1* and pMP220::P_{nodA1}	This study

	vectors	
nodD2-OE pMP220::P_{nodA1}	Δ*nodD1-5* harbouring the pK18*mobsacB*::*nodD2* and pMP220::P_{nodA1} vectors	This study
nodD3-OE pMP220::P_{nodA1}	Δ*nodD1-5* harbouring the pK18*mobsacB*::*nodD3* and pMP220::P_{nodA1} vectors	This study
nodD4-OE pMP220::P_{nodA1}	Δ*nodD1-5* harbouring the pK18*mobsacB*::*nodD4* and pMP220::P_{nodA1} vectors	This study
nodD5-OE pMP220::P_{nodA1}	Δ*nodD1-5* harbouring the pK18*mobsacB*::*nodD5* and pMP220::P_{nodA1} vectors	This study
Mesorhizobium loti R7A DsRED	Wild-type strain harbouring the pFAJDsRed vector	Kelly *et al*., 2013
Rhizobium tropici CIAT 899 DsRED	Wild-type strain harbouring the pFAJDsRed vector	This study
Δ*nodD1* DsRED	*nodD1* deletion mutant harbouring the pFAJDsRed vector	This study
Δ*nodD2* DsRED	*nodD2* deletion mutant harbouring the pFAJDsRed vector	This study
nodD1-LE DsRED	*nodD1*-LE strain harbouring the pFAJDsRed vector	This study
nodD2-LE DsRED	*nodD2*-LE strain harbouring the pFAJDsRed vector	This study
nodD1-OE DsRED	*nodD1*-OE strain harbouring the pFAJDsRed vector	This study
*nodD2-O*E DsRED	*nodD2*-OE strain harbouring the pFAJDsRed vector	This study
Sinorhizobium fredii HH103	Wild-type strain (Rif^R)	Madinabeitia *et al*., 2002
HH103 *nodD2*-OE	HH103 harbouring the pBBR1-MCS-5::*nodD2* vector	This study

Supplementary references

de Maagd RA, Wijffelman CA, Pees ELLY, Lugtenberg BJ. 1988. Detection and subcellular localization of two Sym plasmid-dependent proteins of Rhizobium leguminosarum biovar viciae. J Bacteriol, 170, 4424-4427.

Del Cerro P, Ayala-García P, Buzón P, Castells-Graells R, López-Baena FJ, Ollero FJ, Pérez-Montaño F. 2020. OnfD, an AraC-type transcriptional regulator encoded by *Rhizobium tropici* CIAT 899 and involved in Nod factor synthesis and symbiosis. App Env Microbiol 86, e01297-20.

Kelly SJ, Muszyński A, Kawaharada Y, Hubber AM, Sullivan JT, Sandal N, Carlson RW, Stougaard J, Ronson CW. 2013. Conditional requirement for exopolysaccharide in the *Mesorhizobium-Lotus* symbiosis. Mol Plant-Microbe Interact 26, 319-329.

Kovach ME, Elzer PH, Hill DS, Robertson GT, Farris MA, Roop II RM, Peterson KM. 1995. Four new derivatives of the broad-host-range cloning vector pBBR1MCS, carrying different antibiotic-resistance cassettes. Gene 166, 175-176.

Martínez-Romero E, Segovia L, Mercante FM, Franco AA, Graham P, Pardo MA. 1991. *Rhizobium tropici*, a novel species nodulating *Phaseolus vulgaris* L. beans and *Leucaena* sp. trees. Int J Syst Evol Microbiol 41, 417-426.

Madinabeitia N, Bellogín RA, Buendía-Clavería AM, *et al*. 2002. *Sinorhizobium fredii* HH103 has a truncated nolO gene due to a -1 frameshift mutation that is conserved among other geographically distant *S. fredii* strains. Molecular Plant Microbe Interactions 15, 150-159.

Sambrook J, Fritsch EF, Maniatis T. 1989. *Molecular cloning: a laboratory manual* (No. Ed. 2). Cold spring harbor laboratory press.

Schafer A, Tauch A, Jager W, Kalinowski J, Thierbach G, Puhler A. 1994. Small mobilizable multi-purpose cloning vectors derived from the *Escherichia coli* plasmids pK18 and pK19: selection of defined deletions in the chromosome of *Corynebacterium glutamicum*. Gene 145, 69-73.

Simon R. 1984. High frequency mobilization of gram-negative bacterial replicons by the in vitro constructed Tn5-Mob transposon. Mol Gen Genet 196, 413-420.

Table S2. Primers used in this study.

**Primer name**	**Forward**	**Usage**
nodD1-F	5’- ATAAAGCTTCGATGAATGGGCCGTCCA -3’	Cloning of entire *nodD* gene plus own promoter
nodD1-R	5’- AAAGGATCCGCCGATGTACTCGTCTGCTA -3’	Cloning of entire *nodD* gene plus own promoter
nodD1-F (*q*PCR)	5’- CACGGTCGCTATGCGATTGGTA -3’	RT-*q*PCR
nodD1-R (*q*PCR)	5’- GTCGCGGCCAAATTCGGGAA -3’	RT-*q*PCR
nodD2-F	5’- ATAAAGCTTGTAGGCCATAATGTCCAGA -3’	Cloning of entire *nodD* gene plus own promoter
nodD2-R	5’- AAAGGATCCGCGGCTTTATACTCACCA -3’	Cloning of entire *nodD* gene plus own promoter
nodD2-F (*q*PCR)	5’- AAAGCGTCTGGCAAGGGAAG -3’	RT-*q*PCR
nodD2-R (*q*PCR)	5’- TTTTCGTCGAACAGCTTCGC -3’	RT-*q*PCR
nodD3-F	5’- ATAAAGCTTGAGCTACCTCGACTGCTA -3’	Cloning of entire *nodD* gene plus own promoter
nodD3-R	5’- AAAGGATCCCTACCGCCATGATCACCA -3’	Cloning of entire *nodD* gene plus own promoter
nodD3-F (*q*PCR)	5’- TGAAGCCATGCACGACGACGTC -3’	RT-*q*PCR
nodD3-R (*q*PCR)	5’- AGGCCTGGCATCGAGGAATGGT -3’	RT-*q*PCR
nodD4-F	5’- GAGTCTAGACATGCGCTTTAAAGGTCTTGA -3’	Cloning of entire *nodD* gene plus own promoter
nodD4-R	5’- AGAGGATCCGTCCGGCGTAAACTTTCGGA -3’	Cloning of entire *nodD* gene plus own promoter
nodD4-F (*q*PCR)	5’- CAGCACTGTGCGAAAACGACGC -3’	RT-*q*PCR
nodD4-R (*q*PCR)	5’- CTATTGTGGAGCGCGGGCCATT -3’	RT-*q*PCR
nodD5-F	5’- GAGTCTAGACATGCGGTTCAATGGGCTTGA -3’	Cloning of entire *nodD* gene plus own promoter
nodD5-R	5’- AGAGGATCCGCATGTGCGCAGGACAACAT -3’	Cloning of entire *nodD* gene plus own promoter
nodD5-F (*q*PCR)	5’- CCACGACACCTTCCGTTCACCG -3’	RT-*q*PCR
nodD5-R (*q*PCR)	5’- GTGGCAATCGGCGAAACCATGC -3’	RT-*q*PCR
nodB-F (*q*PCR)	5’- ACAAGGTCGCCAATCACA -3’	RT-*q*PCR
nodB-R (*q*PCR)	5’- GCGCATATATTGCACCGA -3’	RT-*q*PCR
16S-F	5’- GGGCTGGTAAGGTTCTGCGC -3’	RT-*q*PCR
16S-R	5’- GGTGGCGCAGCTAACGCAT-3’	RT-*q*PCR

Supplementary Figure legends

Figure S1. Relative gene expression of *Rhizobium tropici* CIAT 899 *nodD1, nodD2, nodD3, nodD4,* and *nodD5* in a quintuple deletion mutant strain expressing the five *nodD* genes individually at low (LE) or high (OE) levels. (**A**) DNA-DNA hybridization and PCR plots showing deletion of each *nodD* gene in the quintuple deletion mutant strain *ΔnodD1-5*. (**B**) Gene expression of *R. tropici CIAT 899 nodD1*, *nodD2, nodD3, nodD4,* and *nodD5* in WT, OE, and LE strains. Expression levels were measured by RT-qPCR, normalized to 16S, and plotted relative to WT. Plots represent the means (lines), standard deviation (error bars), and individual biological replicates (dots). A one-way ANOVA was used to test for differences between mutants with their corresponding WT with a Dunnett correction for multiple comparisons. ** $P < 0.01$; **** $P < 0.0001$.

Figure S2. Number of white (non-colonized) nodules in *L. japonicus* plants quantified 50 days post-inoculation (dpi) with *R. tropici* CIAT 899 WT; quintuple *nodD* mutant *(ΔnodD1-5); nodD1*-LE, *nodD2*-LE, *nodD3*-LE, *nodD4*-LE, and *nodD5*-LE strains (A). Plots represent the means (lines), standard deviation (error bars), and individual biological replicates (dots). A one-way ANOVA was used to test for differences between mutants with a Tukey correction for multiple comparisons (same letters represent no significant difference at 0.05% level).

Figure S3. Shoot dry weights (SDW) in *Phaseolus vulgaris, Lotus japonicus,* and *Lotus burttii* plants inoculated with WT, *nodD*-LE (**A**) and *nodD*-OE (**B**) strains. SDW and representative photographs of the *P. vulgaris, L. japonicus,* and *L. burtii* plants 30 dpi (*P. vulgaris)* or 50 dpi (*L. japonicus,* and *L. burttii*). Plots represent the means (bars), and standard deviation (error bars). A one-way ANOVA was used to test for

differences between mutants with a Tukey correction for multiple comparisons (same letters represent no significant difference at 0.05% level). Scale bar = 30 cm.

Figure S4. (A). Infection threads of *Lotus burttii* and *Lotus japonicus* plants inoculated with *Mesorhizobium loti* R7A strain harbouring *DsRed* fluorescent marker. Scale bar = 100 µm. **(B)**. Intercellular entry of *L. burttii* plants inoculated with *R. tropici* CIAT 899 WT, single deletion mutant Δ*nodD2*, and quintuple deletion mutant strains expressing *nodD1* or *nodD2* (*nodD1*-LE, *nodD2*-LE, *nodD1*-OE, and *nodD2*-OE) and harbouring *DsRed* fluorescent marker. Scale bar = 200 µm.

Figure S5. Infection threads and fully-colonized nodules of *Lotus japonicus* plants inoculated with different *Rhizobium tropici* CIAT 899 strains. Infection threads (top panel) and fully-colonized nodules (lower panel) of *L. japonicus* plants inoculated with *R. tropici* CIAT 899, single deletion mutant Δ*nodD2*, and quintuple deletion mutant strains over-expressing *nodD1* or *nodD2: nodD1*-OE, and *nodD2*-OE harbouring *DsRed* fluorescent marker. The number of plants with infection threads or nodules is indicated on the top right-hand side of each image and scale bar = 200 µm.

Dataset S1. Nod Factors produced by *Rhizobium tropici* CIAT 899 strains grown in the presence or the absence of *nod* gene inducers.

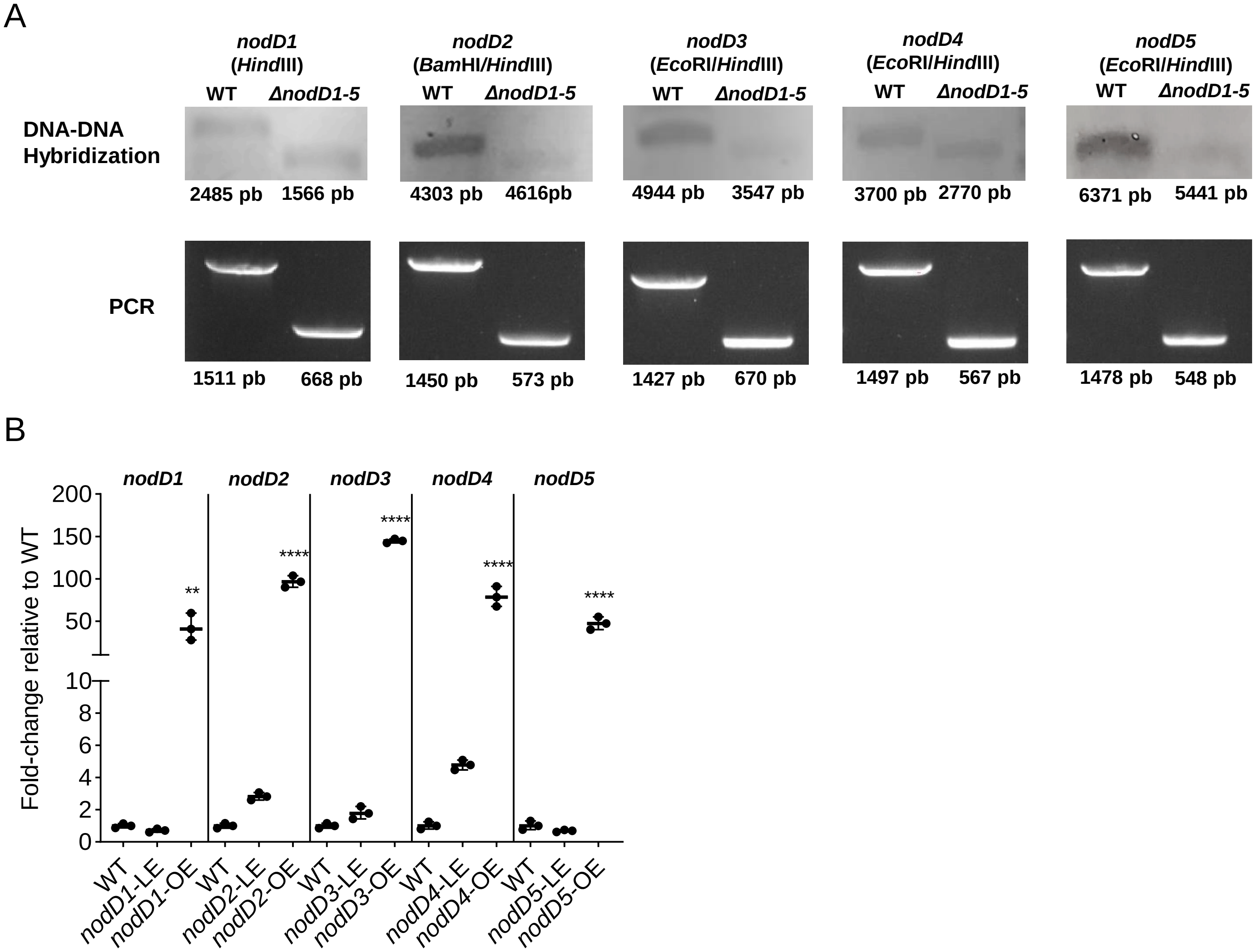

Figure S1. Relative gene expression of *Rhizobium tropici* CIAT 899 *nodD1, nodD2, nodD3, nodD4,* and *nodD5* in a quintuple deletion mutant strain expressing the five *nodD* genes individually at low (LE) or high (OE) levels. (**A**) DNA-DNA hybridization and PCR plots showing deletion of each *nodD* gene in the quintuple deletion mutant strain *ΔnodD1-5*. (**B**) Gene expression of *R. tropici CIAT 899 nodD1, nodD2, nodD3, nodD4,* and *nodD5* in WT, OE, and LE strains. Expression levels were measured by RT-qPCR, normalized to 16S, and plotted relative to WT. Plots represent the means (lines), standard deviation (error bars), and individual biological replicates (dots). A one-way ANOVA was used to test for differences between mutants with their corresponding WT with a Dunnett correction for multiple comparisons. ** $P < 0.01$; **** $P < 0.0001$.

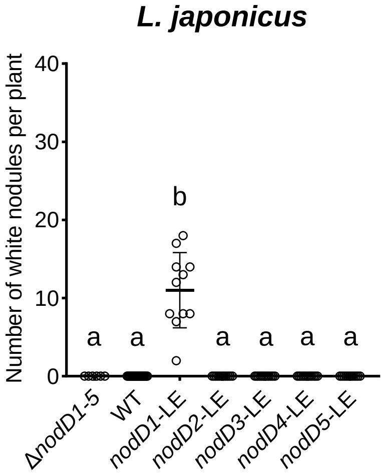

Figure S2. Number of white (non-colonized) nodules in *L. japonicus* plants quantified 50 days post-inoculation (dpi) with *R. tropici* CIAT 899 WT; quintuple *nodD* mutant *(ΔnodD1-5); nodD1*-LE, *nodD2*-LE, *nodD3*-LE, *nodD4*-LE, and *nodD5*-LE strains Plots represent the means (lines), standard deviation (error bars), and individual biological replicates (dots). A one-way ANOVA was used to test for differences between mutants with a Tukey correction for multiple comparisons (same letters represent no significant difference at 0.05% level).

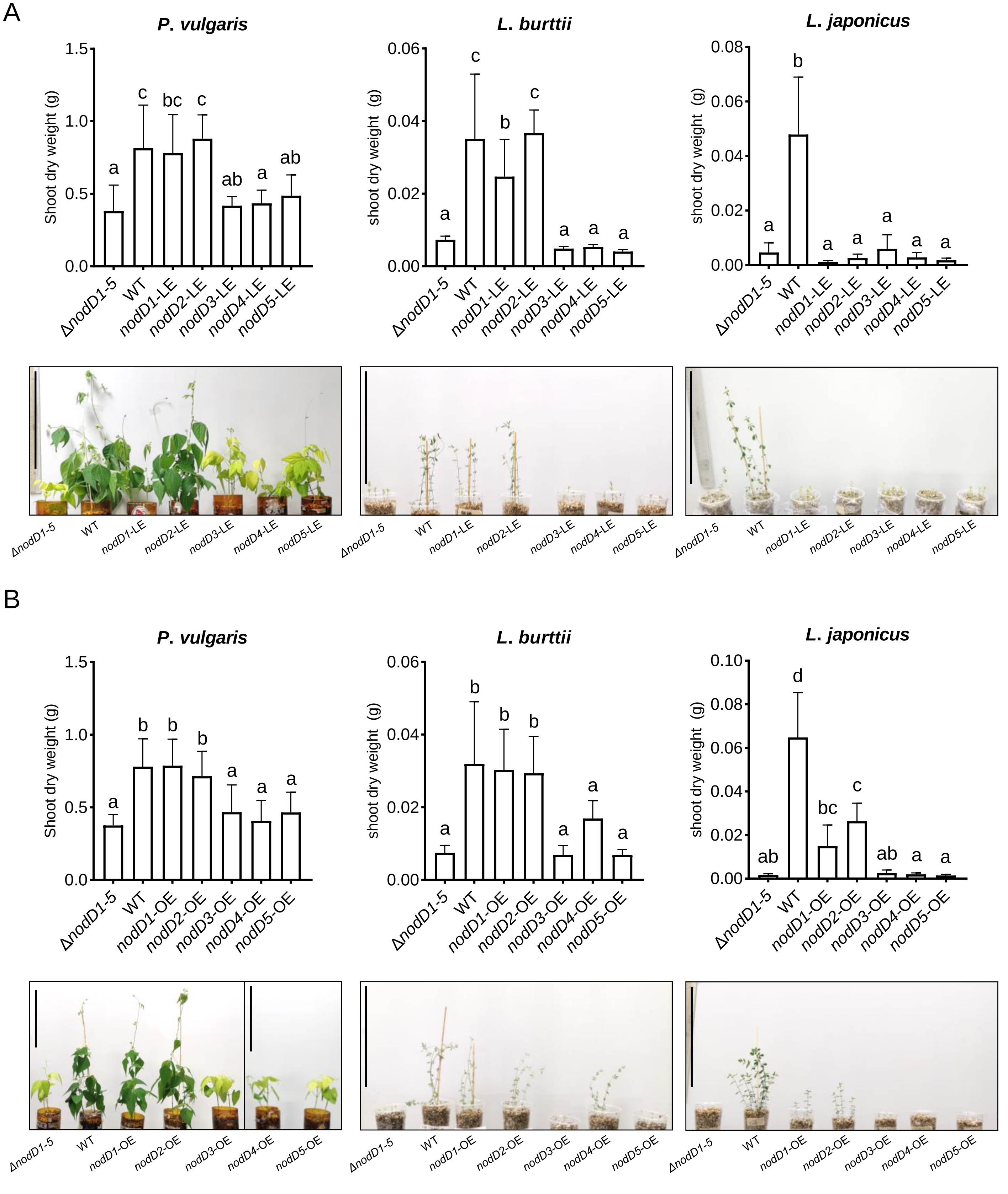

Figure S3. Shoot dry weights (SDW) in *Phaseolus vulgaris, Lotus japonicus,* and *Lotus burttii* plants inoculated with WT, *nodD*-LE (**A**) and *nodD*-OE (**B**) strains. SDW and representative photographs of the *P. vulgaris, L. japonicus,* and *L. burtii* plants 30 dpi (*P. vulgaris)* or 50 dpi (*L. japonicus,* and *L. burttii*). Plots represent the means (bars), and standard deviation (error bars). A one-way ANOVA was used to test for differences between mutants with a Tukey correction for multiple comparisons (same letters represent no significant difference at 0.05% level). Scale bar = 30 cm.

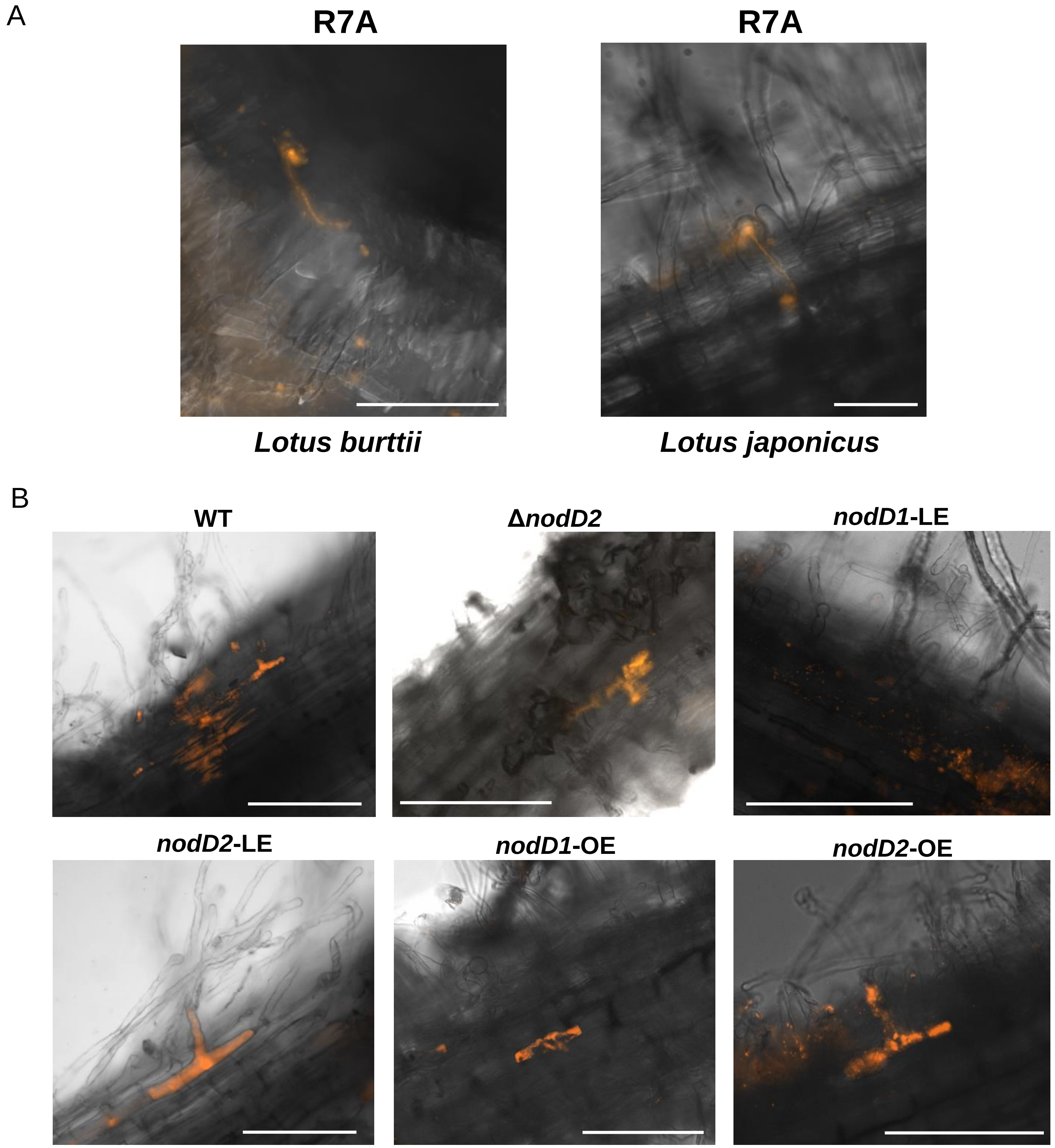

Figure S4. (**A**). Infection threads of *Lotus burttii* and *Lotus japonicus* plants inoculated with *Mesorhizobium loti* R7A strain harbouring *DsRed* fluorescent marker. Scale bar = 100 µm. (**B**). Intercellular entry of *L. burttii* plants inoculated with *R. tropici* CIAT 899 WT, single deletion mutant Δ*nodD2,* and quintuple deletion mutant strains expressing *nodD1* or *nodD2* (*nodD1*-LE, *nodD2*-LE, *nodD1*-OE, and *nodD2*-OE) and harbouring *DsRed* fluorescent marker. Scale bar = 200 µm.

A

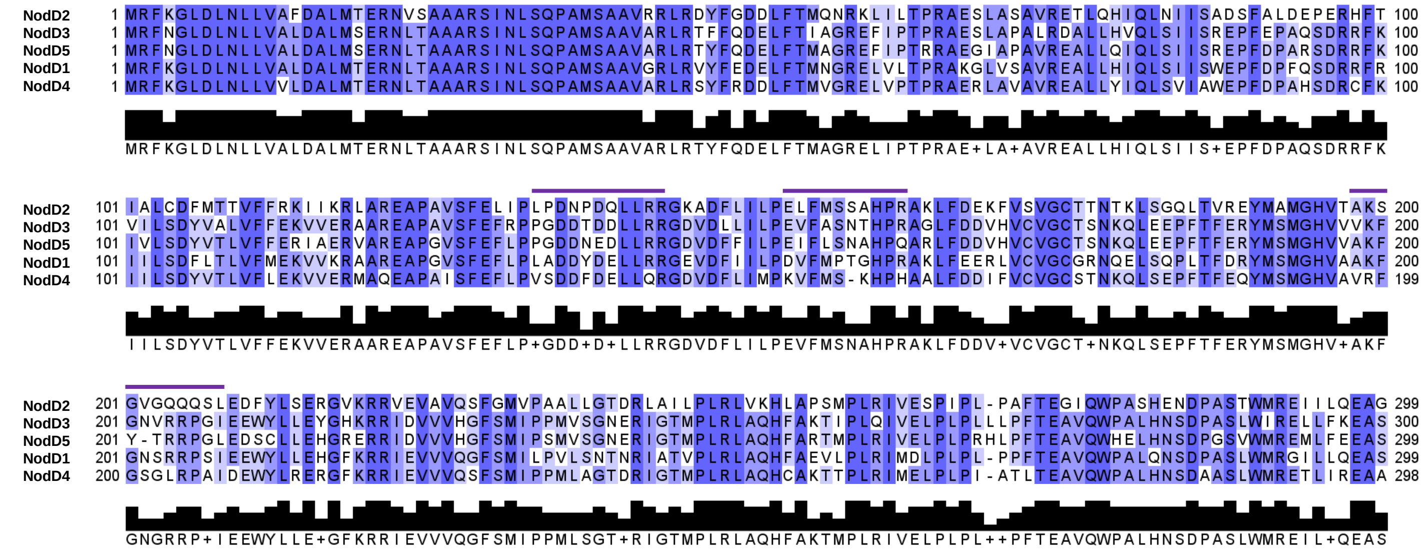

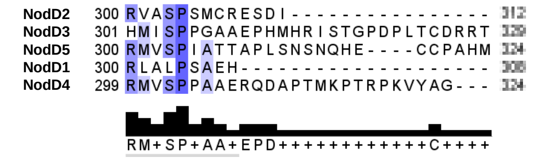

	NodD2	NodD3	NodD5	NodD1	NodD4
NodD2	100	54.81	54.98	61.36	57.23
NodD3	54.81	100	77.78	70.13	69.14
NodD5	54.98	77.78	100	68.08	67.08
NodD1	61.36	70.13	68.08	100	71.01
NodD4	57.23	69.14	67.08	71.01	100

Percentage identity matrix

B

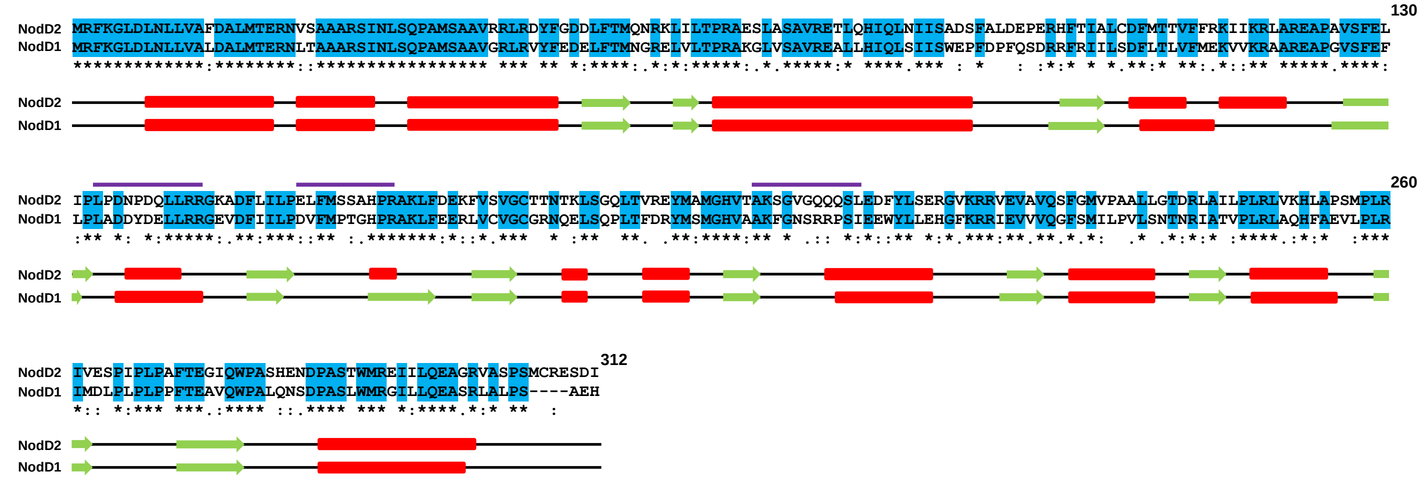

Figure S5. Amino acid alignments of *Rhizobium tropici* CIAT 899 NodD1, NodD2, NodD3, NodD4, and NodD5 proteins. (**A**) Sequence alignment and percentage identity matrix made using Clustal2.1. In sequence alignment, conserved amino acids are highlighted in blue, black bars below represent conserved sequence similarity, and amino acid indicated below represents the most prevalent amino acid. (**B**) Sequence alignment and predicted secondary structure of NodD1 and NodD2. Conserved amino acids are highlighted in blue. Identical amino acid residues are indicated by (*), similar residues by a colon(:), semi-conserved residues by (.) and no residue by a (-). Red bars and green arrows indicate predicted α-helix and β-sheet secondary structures, respectively. Purple lines correspond to the three predicted ligand-binding domains.
